# Phenotypic alteration of macrophages during osteoarthritis: a systematic review

**DOI:** 10.1186/s13075-021-02457-3

**Published:** 2021-04-10

**Authors:** Xiaobo Zhu, Chien-Wei Lee, Hongtao Xu, Yu-Fan Wang, Patrick S. H. Yung, Yangzi Jiang, Oscar K. Lee

**Affiliations:** 1 Institute for Tissue Engineering and Regenerative Medicine, The Chinese University of Hong Kong, Hong Kong, China; 2 Department of Orthopaedics and Traumatology, Faculty of Medicine, The Chinese University of Hong Kong, Hong Kong, China; 3 School of Biomedical Sciences, Faculty of Medicine, The Chinese University of Hong Kong, Hong Kong, China; 4 Li Ka Shing Institute of Health Sciences, Faculty of Medicine, The Chinese University of Hong Kong, Hong Kong, China; 5 Department of Orthopedics, China Medical University Hospital, Taichung, Taiwan

**Keywords:** Osteoarthritis, Macrophage, Innate immunity, Immunomodulation

## Abstract

**Objective:**

Osteoarthritis (OA) has long been regarded as a disease of cartilage degeneration, whereas mounting evidence implies that low-grade inflammation contributes to OA. Among inflammatory cells involved, macrophages play a crucial role and are mediated by the local microenvironment to exhibit different phenotypes and polarization states. Therefore, we conducted a systematic review to uncover the phenotypic alterations of macrophages during OA and summarized the potential therapeutic interventions via modulating macrophages.

**Methods:**

A systematic review of multiple databases (PubMed, Web of Science, ScienceDirect, Medline) was performed up to February 29, 2020. Included articles were discussed and evaluated by two independent reviewers. Relevant information was analyzed with a standardized and well-designed template.

**Results:**

A total of 28 studies were included. Results were subcategorized into two sections depending on sources from human tissue/cell-based studies (12 studies) and animal experiments (16 studies). The overall observation indicated that M1 macrophages elevated in both synovium and circulation during OA development, along with lower numbers of M2 macrophages. The detailed alterations of macrophages in both synovium and circulation were listed and analyzed. Furthermore, interventions against OA via regulating macrophages in animal models were highlighted.

**Conclusion:**

This study emphasized the importance of the phenotypic alterations of macrophages in OA development. The classical phenotypic subcategory of M1 and M2 macrophages was questionable due to controversial and conflicting results. Therefore, further efforts are needed to categorize macrophages in an exhaustive manner and to use advanced technologies to identify the individual roles of each subtype of macrophages in OA.

**Supplementary Information:**

The online version contains supplementary material available at 10.1186/s13075-021-02457-3.

## Introduction

Osteoarthritis (OA) is the most common degenerative joint disorder, mainly affecting the weight-bearing joints such as knees and hips [1], and the non-weight-bearing joints such as the hand and temporomandibular joints [2]. OA is the main cause of physical disability and has been predicted to afflict approximately 67 million people in USA by 2030 [3]. Though risk factors such as aging, obesity, genetic predisposition, and joint trauma have been identified for OA initiation [4–7], few effective treatments are available to prevent OA due to the insufficient understanding of the pathogenesis [8]. Recently, accumulating evidence indicates that the inflammation significantly contributes to OA in addition to the abnormal mechanical loading [9]. OA is gradually viewed as a low-grade inflammatory disease affecting the whole joint besides articular cartilage [10]. Collectively, these findings highlight the profound role of innate immunity in the progression of OA.

As a critical part of the innate immune system, macrophage has long been considered as an important participant in OA. For instance, depletion of synovium macrophages by magnetic beads (anti-CD14-conjugated magnetic beads) [11] or chemicals (e.g., clodronates) [12] contributes to decreasing cartilage catabolic enzymes such as MMP13 and Adamts4. However, the systemic ablation of macrophage in MaFIA (Macrophage Fas-Induced Apoptosis) mice leads to a severe synovitis in obese OA model, implying the complicated roles of macrophages in OA [13].

Macrophages play diverse roles in development, inflammation, and tissue repairing. The plasticity of macrophages enables the cells to make adjustments towards local microenvironments and respond to a wide range of stimuli under both physiological and pathological conditions [14, 15]. Studies in ferreting out the role of macrophages in inflammation have progressed recently. Researchers have identified two different polarization status of macrophages when confronted with different stimuli. In inflammatory phase, classically activated M1 macrophages are recruited and produce high levels of pro-inflammatory cytokines and chemokines, such as tumor necrosis factor-alpha (TNF-α), IL-1, and IL-6. On the other hand, alternatively activated M2 macrophages are needed for the resolution of inflammation. M2 macrophages reverse the inflammatory process by releasing anti-inflammatory factors such as IL-10 and secreting growth factors, such as transforming growth factor-beta (TGF-β) [16]. Therefore, polarization of macrophages at different inflammatory stages might account for various pathological processes. Although the concept of macrophage M1/M2 polarization provides an effective system to study macrophages in vitro, the exact definition and phenotypic transition of macrophages in in vivo studies are still less defined [17].

As mentioned, emerging studies aimed to control inflammation in different diseases by targeting phenotypic changes of macrophages. In pre-clinical models, normalizing the aberrant M1/M2 ratio has been suggested as a therapeutic strategy for macrophage-involved diseases, such as atherosclerosis, lung cancer, and bone diseases including osteoporosis and osteoarthritis [18–22].

In this study, we systematically reviewed recent key findings of macrophage polarization in OA, evaluated the role of phenotypic alterations in macrophages, and summarized the current and potential interventions via the modulations of macrophages.

## Methods

### Search strategy

A systematic search was set up on PubMed, WOS (Web of Science), Ovid (MEDLINE database), Embase (Elsevier Database), and Science Direct (Elsevier Databases), according to the Preferred Reporting Items for Systematic Reviews and Meta-Analyses (PRISMA) guidelines [23]. It was performed without any limitation to the publication date in order to identify all articles on the role of M1 and M2 macrophages in OA. The medical term for osteoarthritis was used in combination with phenotypic alterations of macrophages, which was shown below: osteoarthritis [Title/Abstract] AND macrophage [Title/Abstract] AND (Polarization [Title/Abstract] OR polarization [Title/Abstract] OR M1 [Title/Abstract] OR M2 [Title/Abstract] OR inflammation [Title/Abstract]). The search was last updated on February 29, 2020.

### Screening process

All articles were screened by two independent investigators (XBZ and HTX). The reviewing selection process was based on title, abstract, and full-text level, using a well-established screening tool, Covidence [24].

The eligibility of each article was determined according to the inclusion and exclusion criteria. Studies were included if they met the following criteria: (i) relevant to the searching strategy, (ii) English-written articles within the recent 20 years, and (ii) availability of full-length research articles. Exclusion criteria were present as following: non-English written studies, case studies, review articles, editorials, letters, conference paper, or book chapters. Moreover, the contents of the articles were also taken into consideration. Studies that were not related to OA or without measurements of macrophages were excluded.

Studies meeting all criteria were included, and the quality assessment of these articles was based on heterogeneity and methodological quality. Methodological qualities of included studies were based on the quality systems, which were raised by Wells et al. [25–28]. Details about the qualities of the included studies were listed in Supplemental materials.

At each step of screening, a final consensus was reached after the mutual discussion between two investigators (XBZ and HTX) when different opinions existed. Data were extracted and tabulated by XBZ, and then, a subset of key variables was validated by HTX.

**Fig. 1:**
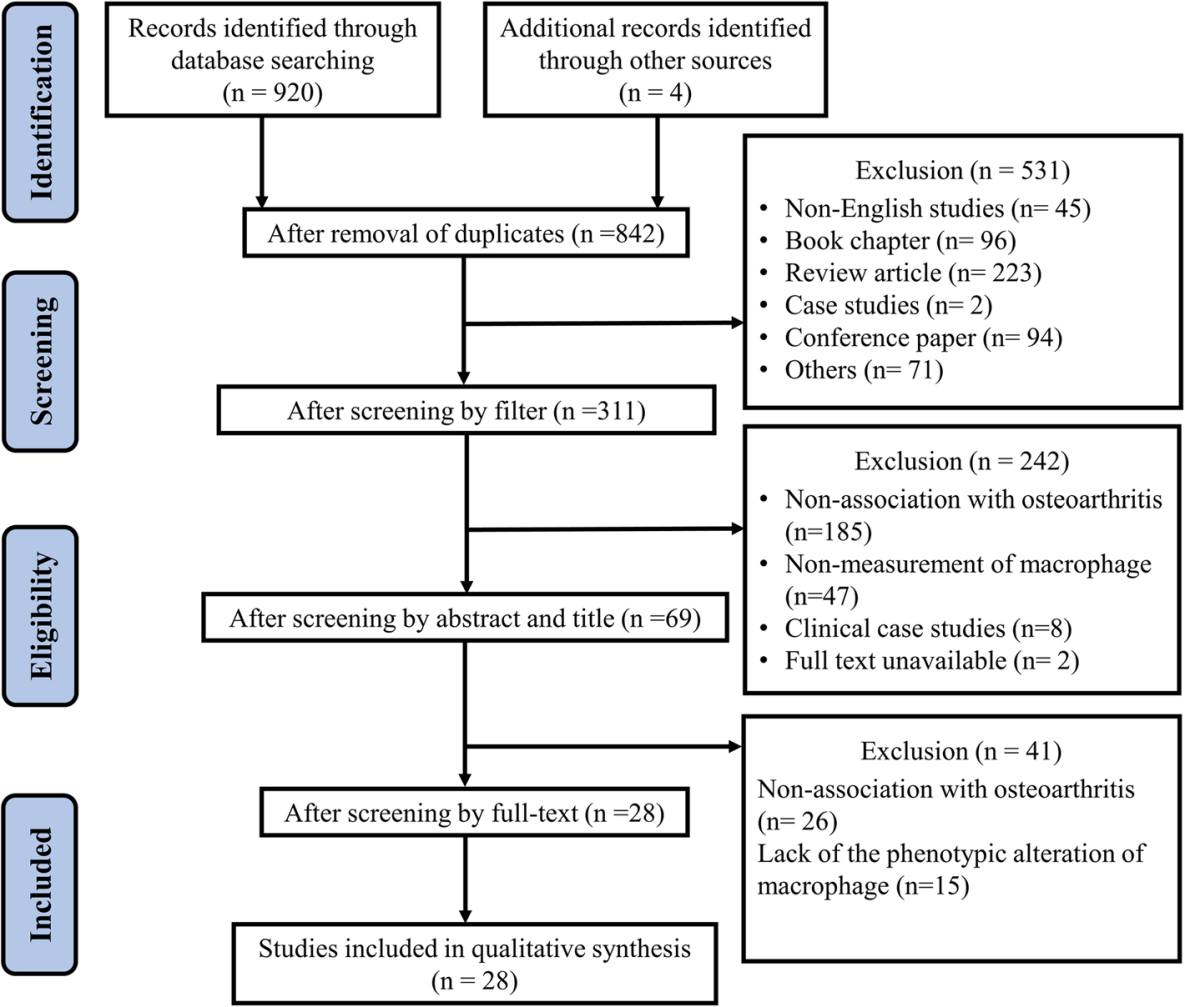
Flowchart of studies included in the systematic review. After the application of all inclusion and exclusion criteria, 28 studies were identified for analysis

All included articles were double-checked by the third investigator (YFW). Similarly, relevant data were extracted and analyzed with a standardized method designed for this review (see Fig. 1. for the flowchart).

## Results

**Fig. 2:**
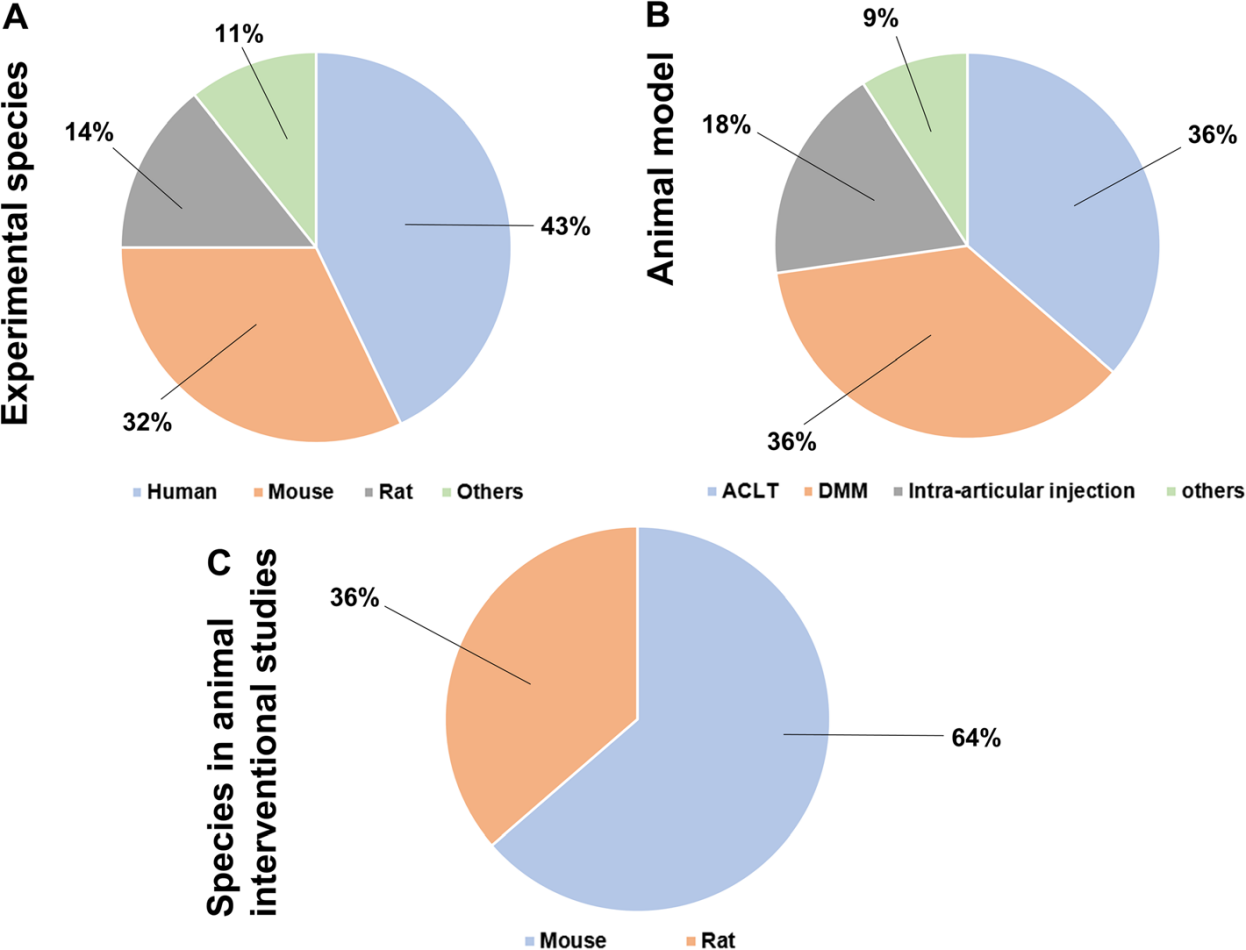
Characteristic outcomes of experimental species, animal models, and species distribution in animal interventional studies. **a** The proportion of experimental species. 43% studies (12/28) were based on primary cells or samples from human. 32% studies (9/28) were based on primary cells or samples from mice. 14% studies (4/28) were based on primary cells or samples from rats. 11% of studies (3/28) were based on samples from canine and equinel; **b** Pie chart illustrating the types of animal models used in the studies. 36% the animal models were ACLT (4/11). 36% the animal models were DMM (4/11). 18% the animal models were intra-articular injection of collagenase (2/11). 9% the animal models were other animal models including osteochondral defect model (1/11); **c** Species distribution in interventional animal studies. 64% the interventional studies were based on mouse models. 36% the interventional studies were based on rat models

Of the 920 articles screened that were considered to be relevant to the research topic, 28 articles were finally included. The screening process is available in Fig. 1. The detailed information of the 28 selected articles are shown in Table 1. In this review, the included articles were categorized into two sections based on the species as different species have different cellular compartments [56]. The distributional proportions of all species in included articles are shown in Fig. 2a. Among all included articles, studies focusing on tissues or cells from human species constituted less than half of total (12/28), followed by animal studies (16/28), including mouse (9/28), rat (4/28) and other animals (canine and equine studies, 3/28). Within these two subcategories (human-derived and animal studies), the origin of macrophages was also evaluated, especially for the peripheral macrophages (peripheral blood monocyte-derived macrophages) and the tissue-resident macrophages (synovial macrophages). In each subcategory, further evaluation was conducted on the interventions that could impact or modulate the M1/M2 ratio in OA progression.

**Table 1:** Characteristics and major conclusion of included studies

Author	Cell source	Species	Study type	Conclusions
Mahon et al. [29]	PBMC	Human	In vitro	BCP promotes macrophage M1 polarization during OA pathogenesis 2DG reverses BCP-induced M1 polarization in OA
Zhou et al. [30]	RAW264.7	Mouse	In vitro *&* In vivo	ACLT model induces an increase of M1 polarization in synovial macrophage. Kin attenuates the number of M1 macrophage and up-regulated the M2 macrophage.
Jablonski et al. [31]	N/A	Mouse	In vivo	The predominant macrophage population observed in uninjured knee joint is M1 macrophage.
Benjamin et al. [32]	N/A	Dog	In vivo	CR (cruciate rupture) model leads to a M1 polarization in synovial macrophage.
Haltmayer et al. [33]	N/A	Horse	In vitro	The osteochondral-synovial explant co-culture OA-model indicates a shift towards M1 phenotype during OA progression
Liu et al. [34]	Synovial fluid; PBMC	Human	*In vivo*	Human knee synovial macrophage displays an increased M1 polarization and decreased M2 polarization.
Sambamurthy et al. [35]	N/A	Mouse	In vivo	DMM model presents an elevated M1 polarization and decreased M2 polarization during OA progression
Wang et al. [36]	BMMC	Mouse	In vivo *&* In vitro	DMM model demonstrates increased numbers of M1 macrophages and decreased number of M2 macrophage. BTZ could reversed this pathological process
Zhang et al. [37]	Synovium	Mouse	In vivo	Both human OA and CIOA model display an elevated M1 polarization
Timur et al. [38]	Hoffa’s fat pad	Human	In vivo	PGE2 released by OA HFP is positively associated with M1 macrophages polarization, indicating a role for macrophages. Celecoxib modulated the inflammation ratio towards a more favorable anti-inflammatory M2 phenotype
Topoluk et al. [39]	Synovium and cartilage explant	Human	In vitro	OA coculture of synovium with cartilage demonstrates increased M1 polarization.
Wu et al. [13]	N/A	Mouse	In vivo	DMM model demonstrates increased numbers of M1 macro
Manferdini et al. [40]	Human SMMC & PBMC	Human	In vitro	ASCs are responsible for the switching of activated-M1-like inflammatory macrophages to a M2-like phenotype
Pal et al. [41]	PBMC	Human	In vitro	SFN could shift monocyte/macrophage differentiation towards the anti-inflammatory M2 type
Siebelt et al. [42]	Human monocyte	Rat	In vitro	TA induces a M2 polarization in macrophage
Fahy et al. [43]	SMMC and fibroblast	Human	In vitro	M1 macrophages downregulate MSC chondrogenesis
Tsuneyoshi et al. [44]	N/A	Human	In vitro	The distribution and M1/M2 expression profiles of synovial macrophages are different between OA and RA synovium.
Zhang et al. [45]	N/A	Rat	In vivo	In a Rat osteochondral defect model, M2 macrophages in cartilage and synovium increase. The intervention of exosomes increases the M2 macrophages and decreases M1 macrophage
Hu et al. [46]	N/A	Rat	In vivo	Quercetin promotes cartilage repair by modulating macrophages polarization to M2 macrophages in Rat OA model
Dai et al. [47]	RAW264.7	Rat	In vivo *&* In vitro	SCII immunomodulates a phenotype shift of macrophages from M0 to M2 during OA progression
Barreto et al. [48]	PBMC	Human	In vitro	Lumican contributes to the innate immune-mediated pathogenesis of primary IOA via macrophage M1 polarization
Kraus et al. [49]	N/A	Human	In vivo	One patient OA synovium presents M1 and M2 marker simultaneously.
Utomo et al. [50]	PBMC	Human	In vitro	Dexamethasone lowers M1/M2 proportion in OA synovium.
Perla et al. [51]	THP-1 cell; PBMC	Human	In vitro	Overexpression of CD163 contributes the transition from M1 to M2 when stimulated with LPS
Nobuaki et al. [52]	N/A	Mouse	In vivo	Polarization towards M2-like macrophages from M1-like macrophages in the synovium is associated with OA alleviation by SRT2104.
Menarim et al. [53]	BMMC	Horse	In vitro	BMNCs cultured in normal synovial fluid or inflamed synovial fluid exhibit aspects of both M1 and M2 phenotypes and immunoregulatory response.
Zhou et al. [54]	RAW264.7	Mouse	In vivo *&* In vitro	Modified Nanoparticles suppress M1 macrophages and upregulate M2 macrophage infiltration in the synovium, thus preventing cartilage degeneration
Shu et al. [55]	N/A	Mouse	In vivo	Hyaluronan could increase the anti-fibrotic M2c macrophages (F4/80^+^CD206^+^CD301^+^) 12 weeks post DMM

Abbreviations: *PBMC* peripheral blood monocytes, *SMM* synovium-derived macrophage, *BMMC* bone marrow mononuclear cells

### Human tissue/cell-based studies

In mammals, most organs contain tissue-resident macrophages. These macrophages are believed to be distributed into different tissues during embryogenesis [15] and are essential for maintaining immune homeostasis. On the other hand, circulating monocyte-derived macrophages also play a crucial role in inflammation. In response to inflammation, circulating monocytes are recruited to inflamed tissues and subsequently differentiate into macrophages in situ by local inflammatory mediators [57]. For healthy individuals, a small number of macrophages constitutively reside in the knee joint. However, in the OA knees, the intensity and infiltrated areas of macrophages in synovium are significantly increased [58].

#### M1 and M2 macrophage in the peripheral blood from OA patients

Studies of peripheral blood monocytes (PBMCs) in OA were carried out in the past few years. PBMCs would partially polarize towards M1 or M2 macrophages after stimuli, and the phenotypic alterations of macrophages were identified based on cellular markers.

In one study, PBMCs were isolated and induced to differentiate into macrophages, and then, CD14^+^CD11b^+^ macrophages (M0, naive macrophages) were purified and selected. Upon stimulated with OA-related metabolite, such as basic calcium phosphate (BCP) crystals, macrophages produced higher levels of chemokines, such as CXCL9 and CXCL10, and increased the expression of M1 surface markers, such as CD86 and CD40 [29]. These results implied that the PBMCs tended towards the M1 phenotype during OA.

In another human cell-based study, the proportion of pro-inflammatory CD11c^+^ macrophages was significantly higher in the circulation of OA patients than that of healthy individuals. In contrast, the number of anti-inflammatory CD206^+^ macrophages was markedly lower in OA patients [34]. These results revealed a higher M1/M2 ratio in the peripheral blood of OA patients.

#### M1 and M2 macrophage in synovium from OA patients

Synovium-resident macrophages were activated in OA and contributed to the principal source of cytokines in OA progression [59].

Researchers have identified the abnormal accumulation and phenotypic alterations of macrophages in OA synovium [44, 49], analogous to those in the peripheral blood. Compared to healthy synovium, OA synovium demonstrated a marked elevation of F4/80^+^ (macrophage marker) cells in both intimal and sublining layers, together with a higher number of iNOS^+^ (M1-like macrophage marker) cells in the intimal lining layer. Conversely, CD206^+^ (M2-like macrophage marker) cells showed only a slight but non-significant decrease in OA synovium [37].

Hoffa’s fat pad (HFP) is another macrophage niche in the knee joints [60]. Timur et al. divided the OA conditions into two groups according to the level of Prostaglandin E2 (PGE_2_) in HFP explant culture medium (100 mg fat pad tissue /ml): high PGE_2_ group (> 25 ng/ml) and low PGE_2_ group (< 25 ng/ml). The HFP from high PGE_2_ OA group showed a 21.4-fold higher inflammation ratio than HFP from low PGE_2_ OA group. Meanwhile, HFP from high PGE_2_ OA group demonstrated 3.7-fold lower gene expression of CD163 (M2 macrophage marker) compared to the HFP in low PGE2 group. These results indicated that the role of macrophage polarization might vary in different OA subtype [38].

#### In vitro human tissue/cell-based study

Although the in vitro data from isolated cells or tissues are not yet ready to be translated into clinical interventions in OA. Several in vitro studies with exogenous treatment still showed potentials to normalize the macrophage polarization and protect against OA. For example, overexpression of CD163 in primary human macrophages by a polyethylenimine nanoparticles grafted with a mannose ligand (Man-PEI) contributed to the transition of macrophages from M1 to M2 after being stimulated with LPS [51]. Pal et al. validated that sulforaphane (10 μM) could skew the differentiation of monocytes (human monocyte cell line: THP1 cells; human primary monocytes) towards the anti-inflammatory M2 type [41]. Another sample is lumican (LUM), a major extracellular matrix glycoprotein in articular cartilage, and its expression was significantly upregulated in OA [48]. LUM contributed to the innate immune-mediated pathogenesis of primary OA via promoting macrophage M1 polarization [48], and this made LUM become a promising therapeutic target for OA. In addition, the human adipose-derived MSCs were reported to be responsible for phenotypic switching from M1 to M2 in human macrophages, accompanied with the decreased secretion of inflammatory cytokines such as TNF-α and IL-6 [40]. These cellular and molecular mechanisms are related to the modification of macrophages in OA, and the treatments have shown therapeutic effects in human in vitro studies.

### Animal studies

Due to the complexity of OA, there are still many unknowns in pathogenesis of OA. For example, the exact trigger for initiating the cartilage degradation is still unknown. Also, the underlying mechanisms that lead to disease maintenance rather than resolution are still poorly understood. Therefore, animal studies become valuable to delineate the underlying mechanisms of the disease and develop novel therapies.

In this section, we reviewed the articles on the phenotypic alterations of macrophages in different species and different animal models. Since there are many different types of OA animal models, the proportion of different OA models applied in the included studies is shown in Fig. 2b.

#### M1 and M2 macrophages in synovium from experimental animals

Destabilization of medial meniscus (DMM)-induced OA, a well-established OA model [61], has been widely used to study the macrophage alterations in OA. Four studies were included with DMM model in this section, and all studies reported a significantly higher number of synovial F4/80^+^ cells post DMM, indicating that the innate immune system was activated during DMM-incurred OA progression. One study drew the conclusion that the number of M1 macrophages (F4/80^+^CD86^+^CD63^−^) was significantly increased in murine synovium 6 weeks after DMM [36]. In another study, synovial F4/80^+^CD11c^+^CD206^+^ cells significantly decreased after DMM for both 4 and 8 weeks [35]. Additionally, another study showed a trend towards more NOS2^+^ cells (M1 macrophages) in the DMM-operated joint than those in contralateral joint [36]. Similar trends in the change of macrophage populations were also found in a more severe OA model, anterior cruciate ligament transection (ACLT)-induced rodent OA model [30, 47].

Furthermore, there were three studies in large animals. One was a canine OA model [32], and two were equine OA models [53, 62]. In the canine study, synovial fluid samples were collected and analyzed, and researchers discovered that ratio of positively stained M1-polarized macrophages (CCR7^+^iNOS^+^CD68^+^ cells) to M2-polarized macrophages (CD163^+^Arg1^+^CD68^+^ cells) was higher in OA group than that in the normal control group [32]. In an ex vivo study, the equine osteochondral-synovial explant co-culture system was facilitated as an OA model. Researchers evaluated the ratio of NO (μM)/urea (μM) as a symbol of pro-inflammatory M1-like macrophages as previously described [63] and concluded that the macrophages underwent a shift towards M1 phenotype during OA progression [33]. Recently, another study demonstrated that bone marrow-derived mononuclear cells, which were viewed as a source of macrophages, displayed an M2-like transcriptional characteristics when stimulating with inflamed synovial fluid in an equine OA synovitis model (0.5 ng LPS injection into radiocarpal joint for 8 h) [53].

It was also noteworthy that though the phenotypic alterations of macrophages during rheumatoid arthritis (RA) had already been discovered recently [64, 65], there was still a knowledge gap in the phenotypic alterations of peripheral blood-derived monocytes or macrophages in OA animal models.

#### Interventions in animal models

**Fig. 3:**
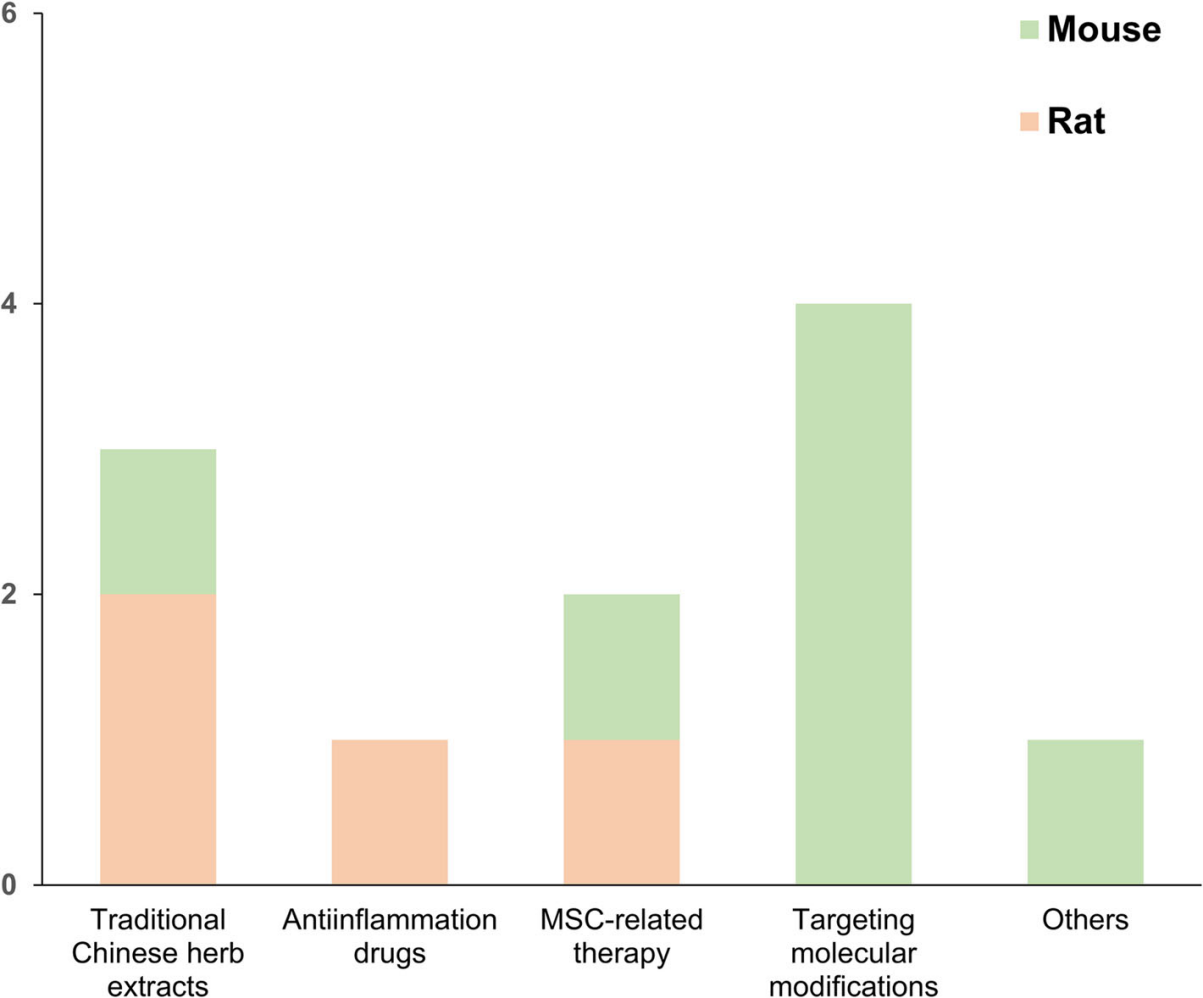
Interventional subcategories. The interventions against OA included traditional Chinese herb extracts, anti-inflammation drugs, MSC-related therapy, targeting molecular modifications, and others

Among all included animal studies, 11 studies were selected and highlighted for the interventions against abnormal changes of macrophages. As shown in Fig. 2c, 64% of these studies (7/11) were based on the mouse models while 36% of the studies (4/11) were based on the rat model. Researchers utilized diverse methodologies to correct the aberrant macrophage polarization (Fig. 3 and Table 2), including interventions regulating and targeting specific signaling pathways [31, 37, 52, 54] and comprehensive interventions such as extracts from traditional Chinese herbs [30, 46, 47], anti-inflammation drugs [42], mesenchymal stem cell (MSC)-related therapies [45, 55], and others [36].

**Table 2:** Details of the interventions in the included studies

Author	Category	Intervention	Detected marker	Interventional site
Jablonski et al. [31]	Endogenous molecule	Cartilage regeneration model; Cartilage regeneration model in CCR2^−/−^CCL2^−/−^ mouse	Cartilage regeneration model: M1: CD38^+^↑; Cartilage regeneration model in CCR2^−/−^CCL2^−/−^ mouse: M2:CD206^+^F4/80^+^↑	Joint
Zhang et al. [37]		Mouse OA model; Rheb1 Knockout (KO) in myeloid cells	Mouse OA model: M0: F4/80↑; M1: iNOS↑; Mouse OA model in Rheb1 KO mouse: M0: (F4/80↑)↓; M1: (iNOS↑)↓; M2: CD206↑	Joint
Zhou et al. [54]		Mouse OA model; Modified ZIF-8 Nanoparticles	Mouse OA model: CD16/32↑; Nanoparticles on OA model: (CD16/32↑) ↓, CD163↑	Joint
Nobuaki et al. [52]		Mouse OA model; SRT2104 (SIRT1 activator)	SRT2104 on OA model: M1: iNOS↓; M2: CD206↑	Joint
Siebelt et al. [42]	Anti-inflammation	Triamcinolone acetonide (TA)	TA: M2: CD163↑	Joint
Zhang et al. [45]	MSC-related option	Exosomes	Rat OA model: M1: CD86↑; M2: CD163↓; Exosome + Rat OA model: (M1: CD86↑) ↓; (M2: CD163↓) ↑	Joint
Shu et al. [55]		Mouse OA model; Bone marrow stem cell (BMSC)	Hyaluronan:M2c (F4/80^+^CD206^+^CD301^+^)↑	Joint
Zhou et al. [30]	Traditional Chinese herb	Kinsenoside (Kin)	Mouse OA model: M1: CD16/32 + ↑; Kin+ Mouse OA model: (M1: CD16/32 + ↑) ↓; M2: CD206↑	Joint
Hu et al. [46]		Quercetin	Quercetin: M2: Arg1↑, MR↑, Ym1↑; Rat OA model: M0: CD68↑; M2: MR↑; Quercetin+ Rat OA model: (M0: CD68↑) ↓; (M2: MR↑) ↑	Joint
Dai et al. [47]		Squid type II collagen (SCII)	SCII: M2: Arg1↑, Ym1↑, MR↑, Fizz1↑; Rat OA model: M0: CD68↑; M2: MR↑; SCII+ Rat OA model: M0:(CD68↑) ↓; M2: (MR↑)↑	Joint
Wang et al. [35]	Others	Mouse OA model Bortezomib (BTZ)	Mouse OA model: M1: F4/80^+^CD86^+^CD63^−^↑; M2: F4/80^+^CD86^−^CD63^+^↓; BTZ+ Mouse OA model: M1: (F4/80^+^CD86^+^CD63^−^↑) ↓; M2: (F4/80^+^CD86^−^CD63^+^↓) ↑	Joint

Noted:

↑ increase, ↓ decrease, *Arg1* Arginase 1, *CCL2* chemokine (C–C motif) ligand 2, *CCR2* chemokine C–C motif receptor 2, *Rheb1* Ras homolog enriched in brain 1

For animal OA models, various traditional Chinese herb extracts such as kinsenoside, quercetin, and squid type II collagen were identified to be able to repolarize the synovial macrophages and attenuate cartilage degeneration in OA [30, 46, 47].

Moreover, to further elucidate the endogenous immunological mechanism of OA, endogenous molecules and the relevant signaling pathways were identified. Mammalian target of rapamycin complex 1 (mTORC1) signaling pathway was reported to be aberrantly activated in synovial macrophages during OA and subsequently contributed to macrophage M1 polarization. These polarized macrophages (M1) produced excessive R-spondin-2 (Rspo2) and then exacerbated experimental OA [37]. By intra-articular injection of Rspo2 neutralizing antibody, the cartilage degeneration incurred by M1 macrophage polarization was effectively ameliorated [37]. In addition, activating silent information regulator 2 ortholog 1 (Sirt1) signaling pathway with a selective Sirt1 activator SRT210 relieved DMM-induced OA, partially through normalizing the synovial macrophage polarization (increased the CD206^+^ M2 macrophages and decreased the iNOS^+^ M1 macrophages) [52]. With the development of nanotechnology, a modified nanoparticle (NP) termed zeolitic imidazolate framework-8 nanoparticle (ZIF-8 NP) was designed to target synovial macrophages and transform macrophage polarization from M1 to M2 phenotype, thus attenuating OA [54]. These interventions and signaling pathways are integrated in Table 3.

**Table 3:** Underlying therapeutic signaling pathway

Signaling pathway	Interventional site
mTORC1-Rheb1/TSC1 [37]	Cartilage
Sirt1 [52]	Cartilage
CCL2-CCR2 [31]	Cartilage
Oxygen and hydrogen peroxide [54]	Synovium

Stem cell-based therapies were also found to relieve OA via modulating macrophage activation [66]. The application of stem cells for cartilage repair relied on their ability to differentiate into chondrocytes and then substitute for the degenerative or dead chondrocytes [67]. Recent studies revealed that the reparative potential of MSCs for OA was also based on its immunological modification on the macrophages [39, 68]. The administration of human embryonic stem cell-derived exosomes in the osteochondral defect model could increase the CD163^+^ macrophages (M2) and decrease the CD86^+^ macrophages (M1) in the joint, and lowered the levels of inflammatory cytokines inside the joint (cartilage and synovium) [68, 69]. Inversely, as a feedback, the inflammatory microenvironment also impacted chondrogenic differentiation of mesenchymal stem cells via macrophage polarization [43].

## Discussion

Recently, the crucial role of synovium resident macrophages in the pathogenesis of RA has been well recognized. Depletion of macrophages or normalizing macrophage phenotype could protect against RA [70–72], and the exact role of M1 and M2 macrophages in RA has also been well reviewed [73]. However, little has been systematically reviewed on the role of macrophages and their polarization in OA. In this study, we systematically reviewed the properties of M1 and M2 macrophages in the periphery, synovial tissue, and synovial fluids of OA patients. We presented important findings from both human and animal OA models and summarized the relevant targeting interventions or comprehensive interventions in these studies, which gave rise to normalizing the phenotype of macrophage (M1 or M2) and alleviating the OA progression.

However, some experimental results were relatively contradictory, which suggest that macrophages have multifaceted and complicated roles in OA. In a DMM-induced OA model in obese mice (high-fat diet for 10 weeks and 20 weeks), Wu et al. eliminated macrophages globally with a small molecule named AP20187 in MaFIA mice. The macrophage-deleted mice immediately exhibited less osteophyte formation following DMM, but these obese mice failed to relieve cartilage deterioration at week 9 and synovial inflammation was also activated in macrophage-depleted mice compared to that in non-depletion mice at week 9 [13]. These observations were inconsistent with previous studies conducted by Blom et al. that deletion of macrophages via intra-articular injection of clodronate liposomes could relieve the inflammatory response and cartilage catabolic enzymes in a collagenase-induced OA model in C57Bl/6 mice [12, 74]. Further, Wu et al. also found that CD3^+^ T cells and neutrophils massively infiltrated into the DMM-operated knee joint and caused severe joint synovitis [13]. The above study emphasized the potential roles of macrophage in maintaining joint homeostasis after injury apart from pro-inflammation and implied that macrophage would participate in limiting the adaptive immune response that developed after the initial innate response. Thus, it was arbitrary to treat OA by merely deleting whole macrophages in the joint, without discriminating the different subtypes of macrophages. Further attention should be paid to identifying and mobilizing the potential positive roles of macrophages on cartilage repair in OA.

Coincidently, Zhang et al. furthered the research on identifying the roles of different phenotypes of macrophages in OA [37]. They demonstrated that M1 but not M2 macrophages accumulated in synovial tissues from human OA and murine collagenase-induced OA. Activating synovial macrophage M2 polarization by ablating an upstream activator of mTORC1 pathway (i.e., Ras homolog enriched in brain 1/Rheb1) in myeloid lineage cells prevented OA development. Conversely, deleting an upstream inhibitor of mTORC1 pathway (i.e., tuberous sclerosis complex 1/TSC1) in myeloid lineage cells enhanced M1 polarization and exacerbated cartilage damage in both surgically induced OA and collagenase-induced OA [37]. Furthermore, they identified R-spondin-2 (Rspo2) as an M1 macrophage-produced protein involved in the subsequent OA progression. Thus, clinical attention could be paid to the mTORC1 signaling pathways in synovial macrophages, and the administration of Rspo2 inhibitor or neutralizing antibody in the knee joints. Based on Zhang’s study, the previous conflicting results (Blom’s and Wu’s studies) that global depletion of macrophages failed to prevent OA might be partially explained. We made the speculation that it was the phenotypic shift (e.g., from M0 to M1 by downregulating TSC1; from M0 to M2 by downregulating Rheb1), other than the number of macrophages that accounted for the conflicting results.

Understanding phenotypic changes of macrophage polarization and the function of each subtype would shed light on the potential clinical implication of the interventions. For instance, regenerative therapies such as human embryonic stem cell-derived exosomes showed potential to prevent cartilage deterioration via activating the pro-survival Akt signaling pathway, and reprogramming joint macrophage by increasing M2 transition and decreasing M1 macrophage infiltration [45]. Intriguingly, a recently developed nanoparticle (modified ZIF-8 NP) showed the similar therapeutic effects on cartilage protection and macrophage repolarization (described in 3.2.2) [54]. Thus, although the macrophage repolarization in OA is still under pre-clinical investigation, it is of great value to attach more importance to regulating macrophage polarization with the respect to the potential clinical application and successful application cases in other skeletal disease such as RA [72, 75].

Emerging improvements regarding the correlation of OA and macrophages have been made these years, but a number of unanswered questions were remained. Firstly, the classical M1/M2 classification was insufficient for researchers to describe and explain the delicate mechanisms, and the subtypes of macrophages were required to be delineated strictly. Although several subtypes of M2 macrophages (e.g., M2a, M2b, M2c, M2d) [76] were categorized based on the in vitro inducing cytokine combinations (i.e., M2a: IL-4 and IL-13; M2b: LPS and immune complex; M2c: IL-10 and glucocorticoids; M2d: IL-6) [77–79]. Researchers still unintentionally overlooked the sub-classification of macrophages in vivo. Only one study focused on the M2c macrophage in OA and concluded that hyaluronan could significantly increase the number of anti-fibrotic M2c macrophages (F4/80^+^CD206^+^CD301^+^) 12 weeks post DMM [55]. Further investigations are thus needed.

The materials and methods in included studies covered the most commonly used molecular biological technologies (shown in Table 2). The phenotypic alterations of macrophage in OA were effectively identified with these technologies, both transcriptionally and translationally. However, the detective methodologies and cellular markers of M0, M1, and M2 macrophages varied and had limitations, and impacted the data interpretation among studies and research models. For instance, the F4/80 was widely adopted as a cellular marker for macrophage in many included studies, but the specify and sensitivity is not that satisfying [80]. The expression of F4/80 varied among mouse mononuclear populations, being very low in the bone marrow Ly6c^+^ monocyte-emanated macrophage, but high in various tissue-resident macrophage derived from fetal yolk sac, such as microglia (brain) and Kuffer cells (liver) [80]. We summarized the cellular markers employed in the included studies in Table 4. In the future, the booming development of single-cell methodologies will help to identify the individual cell types at a single-cell level, which could be used to target distinct macrophage populations precisely.

**Table 4:** Summary of the detected cell surface marker of M0, M1, and M2 macrophage

	Cell surface marker
M0 macrophage	CD68; F4/80
M1 macrophage	CD86; CD40; iNOS/NOS2; CCR7; CD11c; CD16/32
M2 macrophage	CD163; MRC; CD206; Arg1; CCL22

Abbreviation: *CCR7* CC-chemokine receptor 7, *Arg1* Arginase1, *CCL22* C–C motif chemokine ligand 22, *iNOS* inducible NO synthase, *NOS2* NO Synthase2

Although standardization of M1/M2 phenotype gave researchers a uniform framework to study macrophages in vitro and made results from different studies comparable, several drawbacks were still concerned. The rigid subdivision of macrophage as M1 and M2 hindered the understanding of macrophage plasticity in vivo, since classical M1 and M2 polarization were two distinct macrophage subtypes and they were unlikely to occur in a tissue context. For instance, the classical M1/M2 paradigm failed to explain the different transcriptomic changes of human monocyte-derived macrophages and lung-resident alveolar macrophages after stimulated with LPS/IFN-γ (M1 inducer) or IL-4/IL-13 (M2 inducer) [81]. These macrophage responses indicated that there remained room for macrophages to be further grouped. Avraham et al. conducted a single-cell RNA-sequencing to show that peritoneal macrophages in the same microenvironment respond differently to the stimulus of *salmonella* strains that differed by a single gene termed PhoP [82].

The classical concept of the M1/M2 paradigm might be misleading, especially at early stage of inflammation, when tissue-resident and monocyte-derived macrophages coexisted in the local microenvironment. Tissue-resident macrophages expressed relatively higher levels of “M2-like” markers in comparison with mature monocyte-derived macrophages [83, 84]. Without lineage tracing markers, the simultaneous presence of macrophage population in early inflammation (immature monocyte-derived macrophages and few mature tissue-resident macrophages) would be regarded as an “M1-like” polarization tendency due to more immature monocyte-derived macrophages in the mixed population. During weeks-long resolution, monocyte-derived macrophages gradually matured and their phenotype resembled the tissue-resident macrophages. Therefore, the mixed cellular population (mature monocyte-derived and mature tissue-resident macrophages) would display a “shift” towards an “M2-like” phenotype [84]. Thus, in the in vitro study conducted by Menarim et al. [53], we speculated that stimuli (SF or ISF) might fail to mobilize the monocyte-derived macrophages from circulation and incur the insufficiency of the M1-like macrophage.

Recently, Culemann et al. found that in healthy murine and human knee joint, a thin layer of synovium-resident macrophage formed a barrier-like structure, covering the sublining layer of synovium. Disrupting this structure by genetic depletion or pharmacological inhibition of these barrier-forming macrophages exacerbated arthritis progression. Further tracing of these macrophages revealed that these CX3CR1^+^ lining macrophages were originated from a subtype of synovium-resident interstitial macrophage, instead of monocytes [65]. Although these results were discovered in RA model, it also implied that the critical roles of the synovium-resident macrophage or subchondral bone-resident macrophage is worthy of investigation in OA. They were also the potential cellular targets for further therapeutic design. The recently emerging applications of single-cell RNA sequence or mass cytometry could dissect the exact macrophage phenotypes more than M1/M2 paradigm [57, 85].

### Limitation

The quality of included studies was limited due to the low number of high-level evidence. As mentioned in the discussion, the majority of included studies exerted classical methodologies, thus lacking deep insights into the phenotypic alterations of different subtypes of macrophages.

Secondly, the spontaneous OA models were not included due to lack of reports. For instance, aging model in Guinea pig recapitulates the most common cause of human OA [86]. Researches using this model will enhance the translational value in future study.

Thirdly, due to lack of standardized and well-recognized marker for identifying different subtypes of macrophages, the results of different studies were not very comparable.

Lastly, the sample size included in our study was small, both in the number of articles included and the number of animal samples used in some included studies. The small sample size made it at the risk of bias and difficult to reach general conclusions.

## Conclusion

In summary, we reviewed the current studies of phenotypic alterations of macrophages in OA and emphasized the disequilibrium of M1 and M2 macrophage during OA, and potential therapies that could rebalance between M1/M2 macrophages. With a more thorough understanding of macrophages and the improvement in detecting methodology, the detailed macrophage subtypes and their individual roles in OA pathogenesis should be further elucidated.

## Supplementary Information

**Additional file 1: Supplemental 1a.** Methodological quality assessment protocol 1 used for included animal studies (the number of “yes” answers was counted for each study to give a total score out of 8). **Supplemental 1b.** Quality assessment for systemic reviews in experimental animal studies. **Supplemental 2.** Risk of Bias Assessment for in vitro studies according to GRADE Criteria

## Data Availability

All data generated or analyzed during this study are included in this published article.

## Abbreviations

ACLT: Anterior cruciate ligament transection
BCP: Basic calcium phosphate
BMMC: Bone marrow mononuclear cells
DMM: Destabilization of medial meniscus
HFP: Hoffa’s fat pad
IL-1: Interleukin-1
IL-6: Interleukin-6
Lum: Lumican
MaFIA: Macrophage Fas-Induced Apoptosis
mTORC1: Mammalian target of rapamycin complex 1
NP: Nanoparticle
OA: Osteoarthritis
PBMC: Peripheral blood monocytes
PRISMA: Preferred Reporting Items for Systematic Reviews and Meta-Analyses
RA: Rheumatoid arthritis
Rheb1: Ras homolog enriched in brain 1
Rspo2: R-spondin-2
PGE_2_: Prostaglandin E2
Sirt1: Silent information regulator 2 ortholog 1
SMM: Synovium-derived macrophage
TGF-β: Transforming growth factor-beta
TNF-α: Tumor necrosis factor-alpha
TSC1: Tuberous sclerosis complex 1
WOS: Web of Science
