# Supplementary material for: Phenotypic alteration of macrophages during osteoarthritis: a systematic review
Source: Arthritis Res Ther. 2021 Apr 10;23:110. doi: 10.1186/s13075-021-02457-3 (PMC8035781; doi:10.1186/s13075-021-02457-3)
Supplement: Supplementary file 1 — Additional file 1: Supplemental 1a. Methodological quality assessment protocol 1 used for included animal studies (the number of “yes” answers was counted for each study to give a total score out of 8). Supplemental 1b. Quality assessment for systemic reviews in experimental animal studies. Supplemental 2. Risk of Bias Assessment for in vitro studies according to GRADE Criteria [file 13075_2021_2457_MOESM1_ESM.docx]

**Supplemental materials.**

Supplemental 1a. Methodological quality assessment protocol[1] used for included animal studies (the number of “yes” answers was counted for each study to give a total score out of 8).

| Section and topic | NO. | Criteria | Yes (Y)/No (N) |
| --- | --- | --- | --- |
| Title/Keywords/Introduction | (1) | Were the study hypothesis/aim/ objective clearly described？ |  |
| Methods | (2) | Was the animal model for the study well-described？ |  |
|  | (3) | Was the method well-described？ |  |
|  | (4) | Were the data collected time points clearly define？ |  |
|  | (5) | Were the main outcome measures clearly define？ |  |
|  | (6) | Was the experiment group well compared with the control group？ |  |
| Results/Discussion | (7) | Were the results well-described？ |  |
|  | (8) | Were the articles discussed the limitation？ |  |

Supplemental 1b Quality assessment for systemic reviews in experimental animal studies

| Reference | Title/Keywords/Introduction | Methods | | | | | Results/Discussion | |  |
| --- | --- | --- | --- | --- | --- | --- | --- | --- | --- |
|  | (1) | (2) | (3) | (4) | (5) | (6) | (7) | (8) | Total |
| Zhou et al. [47] | Y | Y | Y | Y | Y | Y | Y | N | 7 |
| Jablonski et al. [56] | Y | Y | Y | Y | Y | Y | Y | N | 7 |
| Benjamin et al. [49] | Y | Y | Y | Y | Y | Y | Y | Y | 8 |
| Sambamurthy et al. [46] | Y | Y | Y | Y | Y | Y | Y | Y | 8 |
| Wang et al. [45] | Y | Y | Y | Y | Y | Y | Y | Y | 8 |
| Zhang et al. [37] | Y | Y | Y | Y | Y | Y | Y | Y | 7 |
| Wu et al. [13] | Y | Y | Y | Y | Y | Y | Y | Y | 8 |
| Siebelt et al. [60] | Y | Y | Y | Y | Y | Y | Y | N | 7 |
| Zhang et al. [61] | Y | Y | Y | N | Y | Y | Y | Y | 7 |
| Hua et al. [63] | Y | Y | Y | Y | Y | Y | Y | Y | 8 |
| Dai et al. [48] | Y | Y | Y | N | Y | Y | Y | Y | 7 |
| Nobuaki et al. [57] | Y | Y | Y | Y | Y | Y | Y | Y | 8 |
| Zhou et al. [58] | Y | Y | Y | N | Y | Y | Y | N | 6 |
| Shu et al. [62] | Y | Y | Y | Y | Y | Y | Y | Y | 8 |

The information of No. 1-8 is listed in Supplemental 1a.

Supplemental 2. Risk of Bias Assessment for in vitro studies according to GRADE Criteria

| Reference | Study limitation | Inconsistency | Indirectness | Imprecision | Publication bias | Dose effect | Overall |
| --- | --- | --- | --- | --- | --- | --- | --- |
| Mahon et al. [32] | x | √ | √ | √ | √ | √ | +++ |
| Haltmayer et al. [53] | x | √ | √ | √ | √ | √ | +++ |
| Liu et al. [33] | √ | √ | √ | √ | √ | x | +++ |
| Timur et al. [39] | √ | √ | √ | √ | √ | x | +++ |
| Topoluk et al. [67] | √ | √ | √ | √ | √ | √ | ++++ |
| Manferdini et al. [43] | x | √ | √ | √ | √ | √ | +++ |
| Utomo et al. [86] | √ | √ | √ | √ | √ | √ | ++++ |
| Pal et al. [41] | x | √ | √ | √ | √ | √ | +++ |
| Melle et al. [69] | x | √ | √ | √ | √ | √ | +++ |
| Tsuneyoshi et al. [36] | √ | √ | x | √ | √ | √ | +++ |
| Barreto et al. [42] | √ | √ | √ | √ | √ | √ | ++++ |
| Kraus et a l. [35] | √ | √ | √ | √ | √ | √ | ++++ |
| Perla et al. [40] | x | √ | √ | √ | √ | √ | +++ |
| Menarim et al. [51] | √ | √ | √ | √ | √ | √ | ++++ |

GRADE factors: √: without serious limitations; x: with serious limitations; Unclear, unable to rate items based on available information for Overall Quality of Evidence: + very low; ++ low; +++ moderate; ++++ high

**Reference**

1. Hong-Tao X, Chien-Wei L, Yan LM, Yu-Fan W, Shu-Hang Patrick Y, Kuang-Sheng Oscar L. The shift in macrophages polarization after tendon injury: A systematic review. J Orthop Transl [Internet]. Elsevier Ltd; 2019;21:24–34. Available from: https://doi.org/10.1016/j.jot.2019.11.009
